# Non-operative treatment of metacarpal fractures and patient-reported outcomes: a multicentre snapshot study

**DOI:** 10.1007/s00068-024-02659-9

**Published:** 2024-09-23

**Authors:** L. E. M. de Haas, P. A. Jawahier, T. C. C. Hendriks, D. A. Salentijn, B. T. van Hoorn, R. H. H. Groenwold, N. W. L. Schep, M. van Heijl

**Affiliations:** 1 Diakonessenhuis, Utrecht, Netherlands; 2 Maasstad Ziekenhuis, Rotterdam, Netherlands; 3 Radboud University Nijmegen Medical Centre, Nijmegen, Netherlands; 4 Onze Lieve Vrouwe Gasthuis, Amsterdam, Netherlands; 5 Leiden University Medical Center, Leiden, Netherlands; 6 University Medical Center Utrecht, Utrecht, Netherlands

**Keywords:** Metacarpal fractures, Hand fractures, Non-operative treatment, Patient-reported outcomes

## Abstract

**Purpose:**

This study aimed to investigate practice variation in non-operative treatment methods and immobilisation duration for metacarpal fractures, and to evaluate patient-reported outcomes.

**Methods:**

Conducted in 12 Dutch hospitals over three months in 2020, this study included adult patients with non-operatively treated solitary metacarpal fractures. Fractures were classified into intra-articular base, extra-articular base, shaft, neck, and intra-articular head fractures. The treatment methods (functional treatment allowing digit mobilisation or immobilisation) and immobilisation duration were assessed. Patient-reported outcomes were evaluated using the Michigan Hand Outcomes Questionnaire (MHQ) at three months post-trauma.

**Results:**

Of 389 included patients, shaft fractures were most common (n = 150, 39%), with 93% immobilised, followed by fifth metacarpal neck fractures (n = 93, 24%), with 75% immobilised. Immobilisation rates for fifth metacarpal neck fractures varied between hospitals, ranging from 29% (95% CI 0.10–0.58) to 100% (95% CI 0.78–1.00). The median immobilisation duration for all fractures was 23 days (IQR: 20–28), and hospital setting was independently associated with this duration. Patients with metacarpal shaft fractures immobilised for less than 21 days had higher MHQ scores compared to those immobilised for 21 days or more (median (IQR) 83 (76–100) versus 71 (57–89), p = 0.026).

**Conclusions:**

The results showed practice variation in the treatment of metacarpal fractures, especially in the treatment of fifth MC neck fractures, with some hospitals following the Dutch guideline that advocates functional treatment while others did not. There are suggestions that prolonged immobilisation of metacarpal shaft fractures may lead to a worse MHQ score. These findings underscore the need for adherence to treatment protocols and emphasize functional treatment to potentially improve patient outcomes and cost-effectiveness.

**Supplementary Information:**

The online version contains supplementary material available at 10.1007/s00068-024-02659-9.

## Introduction

Fractures of the metacarpals are common injuries and represent 31–33% of all hand fractures [1, 2]. Metacarpal fractures predominantly affect the young working population and are associated with an economic burden due to resource and productivity costs [3].

Metacarpal fractures can be classified as intra-articular base, extra-articular base, shaft, neck and intra-articular head fractures. Surgical treatment should be considered if there is a rotational deformity leading to symptomatic scissoring, an apex dorsal angulation leading to pseudo-clawing, or shortening with a symptomatic extension lag. Most of these fractures, however, can be treated non-operatively [4]. Static casting in the intrinsic position has traditionally been the preferred treatment. The last decade there is a tendency to use functional treatment (i.e. where mobilization of the fingers and wrist is allowed) with a dynamic splint such as a Lucerne Cast or a pressure bandage [4–8]. Different strategies of non-operative treatment can possibly result in practice variation, which in turn may influence functional and patient-reported outcomes.

An evaluation of current non-operative management for MC fractures could be useful for optimizing patient care and improving outcomes. Therefore, the aim of this multicentre snapshot study was to assess the non-operative treatment methods and duration of immobilisation for metacarpal fractures and to identify potential practice variation. Additionally, we evaluated patient-reported outcomes in relation to non-operative treatment methods and duration of immobilisation.

## Methods

### Study design

A multicentre, observational, snapshot study was performed from August to October 2020. Twelve hospitals in the Netherlands participated including one academic hospital, 10 teaching hospitals and one non-teaching hospital.

The study was reported according to the Strengthening the Reporting of Observational Studies in Epidemiology (STROBE) statement [9]. Local investigators were trained by the coordinating investigator to identify eligible patients and to collect data. Castor Electronic Data Capture software was used to store this data. Ethical approval was obtained from the local ethics committee of all participating hospitals. A waiver of informed consent was granted for this study due to the study design, the expected large study population and associated burden to obtain informed consent for this type of injury, and the minimal risk to human subjects.

### Participants

**Fig. 1:**
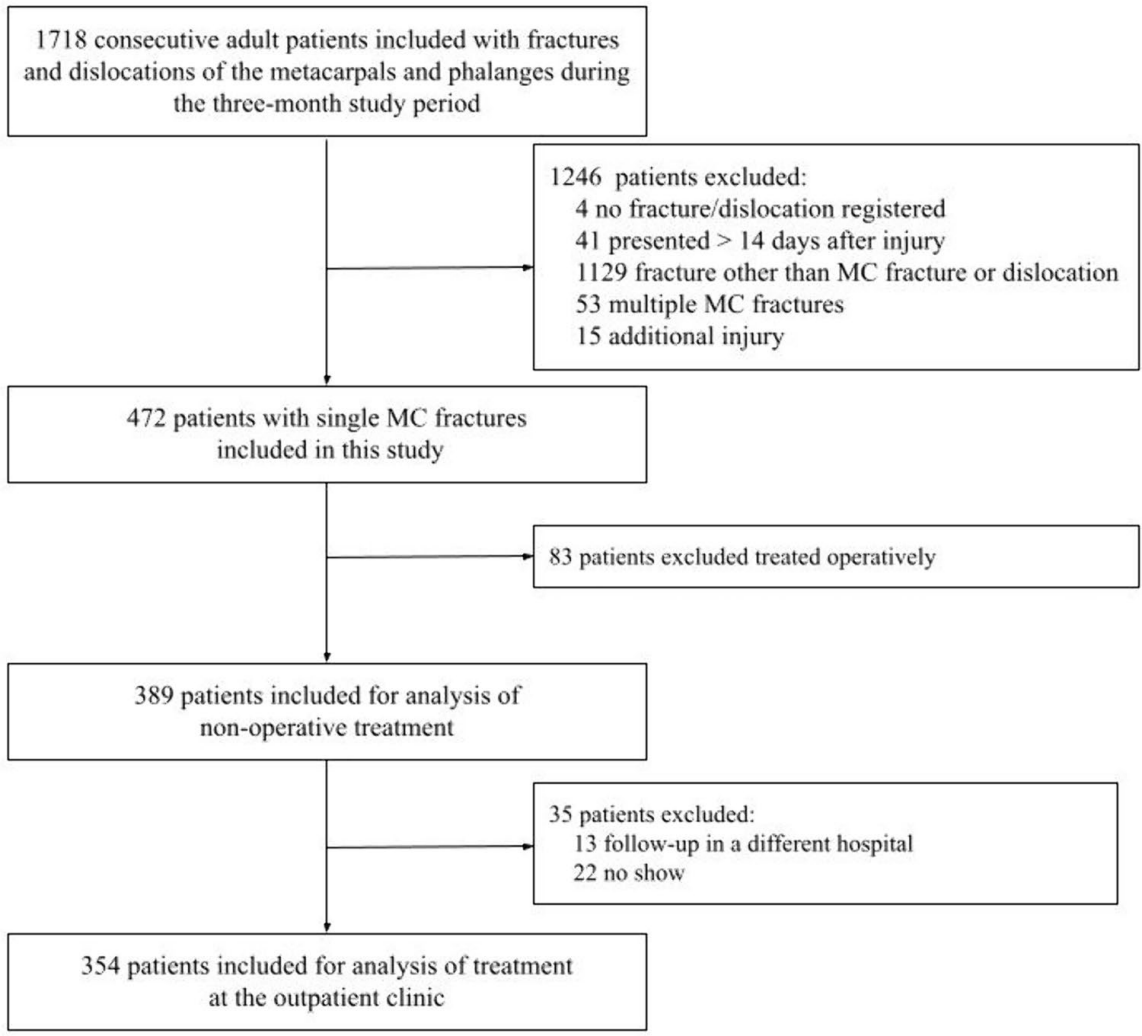
Flow-diagram of included patients

1718 consecutive patients of 18 years and older who presented with fractures or dislocations of the metacarpals and phalanges were included in the database. A selected group of patients from this database were included for this study. Inclusion criteria were patient presentation at the emergency department (ED) within 14 days after injury, no additional injury and a single MC fracture treated non-operatively. The study flow chart is presented in Fig. 1.

### Study variables

Patient demographics were retrieved from the electronic patient file. The following patient and injury characteristics were documented: age, sex, smoking, diabetes mellitus, work status, presence of hand comorbidity, mechanism of injury, dominant hand is affected hand, days between injury and presentation, affected digit, presence of soft tissue injuries (including open fractures, significant lacerations, ligament-, tendon- and nail bed injuries), clinically observed angulation observed in the ED (including volar, dorsal, ulnar or radial angulation), rotational deformity observed in the ED, fracture displacement of more than two millimetres on the radiograph (i.e. an abnormal position of the distal fracture fragment in relation to the proximal bone), closed reduction of the fracture, treating physician (emergency physician trauma, orthopaedic, plastic or surgeon) and referral for hand therapy. Patients who were treated by an emergency physician received definitive treatment at the ED and were not assessed by another medical specialist. Complications were recorded until one month after first presentation.

### Fracture and treatment classification

Fractures were classified as intra-articular base, extra-articular base, shaft, neck and intra-articular head fractures. The classification of fractures was in accordance with the standard radiology report. Uncertainties in the radiologist’s report were addressed through consultation with the local surgeon participating in the collaborative study group.

Non-operative treatment was classified as functional treatment or immobilisation. Functional treatment included all patients who received no immobilisation, a buddy tape, a lucerne cast (a metacarpal cast, leaving the wrist joint and the palmar metacarpophalangeal joint (MCPJ) exposed, and dorsally fixating the proximal interphalangeal joints (PIP) and the MCPJs in 70°–90° of flexion to allow motion of the PIPJs), a removable wrist brace, a removable cast or a pressure bandage. Immobilisation included immobilisation with a cast in neutral position, a cast in intrinsic position or an immobilizing rigid digit splint.

Analyses of treatment method and duration of immobilisation were performed separately for different groups. Specifically, intra-articular and extra-articular base fractures were analysed separately for digits 2 and 3, and for digits 4 and 5. For neck fractures, the results were analysed for digits 2–4, and separately for digit 5. Shaft fractures and intra-articular head fractures were analysed collectively across all digits. Consequently, the analyses were performed for eight distinct groups.

### Primary and secondary outcomes

The primary outcome was the applied non-operative treatment method, i.e. the number of patients who received immobilisation versus functional treatment, and in case of immobilisation the duration of immobilisation. The applied non-operative treatment method was determined at the ED. If variation was observed, a bivariate analysis was performed of the fracture characteristics between functional treatment and immobilisation. This was done for extra-articular base fractures of digit 4 and 5, shaft fractures, and fifth MC neck fractures.

A sub-analysis was performed to compare the percentage of immobilisation between the 12 participating hospitals. This analysis was feasible only for shaft fractures and fifth MC neck fractures. The small number of fractures in the other fracture categories (less than 24 fractures per fracture category) and the limited variation in treatment precluded similar comparative analyses.

Of patients who were eligible for evaluation of follow-up at the out-patient clinic, a change in treatment from immobilisation to functional treatment, and the duration of immobilisation was assessed. Patients who received follow-up in a different hospital and patients who did not attend to the scheduled follow-up appointment were excluded from these analyses.

The secondary outcome was the Michigan Hand Outcomes Questionnaire (MHQ) score [10]. This is a hand specific outcome instrument that contains six distinct scales: (1) overall hand function, (2) activities of daily living, (3) pain, (4) work performance, (5) aesthetics and (6) patient satisfaction with hand function. The maximum score is 100 points, which is the best possible score. For shaft fractures and fifth MC neck fractures, differences in MHQ scores between functional treatment and immobilisation and differences in MHQ scores between an immobilisation period of less than 21 days and more than 21 days were analysed.

### Statistical methods

Baseline characteristics and outcome measures were described using descriptive statistics. For continuous data, the mean and standard deviation (normally distributed data) or the median and percentiles (non-normally distributed data) were reported. For categorical data numbers and frequencies were reported. Visual inspection of the distribution was used to assess normality. Associations between duration of immobilisation and hospital and injury characteristics were analysed using multivariable linear regression analysis, in order to quantify and distinguish the roles of patient characteristics from hospital variation. Expert-based variable selection was used to select independent variables for the analysis. Missing values were imputed by assuming the absence of the outcome (four cases had on clinically observed angulation and three on clinically observed rotational deformity). A p value of < 0.05 was taken as a threshold of statistical significance and all tests were two-sided.

## Results

### Patient characteristics

A total of 389 patients were included with a median age of 36 years (interquartile range (IQR): 26–56), of whom 328 (69%) were men. MC shaft fractures were most common (n = 150, 39%), followed by MC neck fractures (n = 148, 38%). In Table 1, patient characteristics are presented, stratified by the five fracture categories. Complications occurred in five patients (1.3%); one cast related complication, two re-dislocations after initial successful closed reduction requiring surgical treatment, one secondary dislocation requiring surgical treatment, and one asymptomatic malunion.

**Table 1:** Patient and injury characteristics by fracture type

Total	Overall N = 389	Intra-articular base N = 37	Extra-articular base N = 48	Shaft N = 150	Neck N = 148	Intra-articular head N = 6
Median age in years (IQR)	36 (25, 56)	41 (28, 63)	42 (27, 68)	38 (25, 56)	32 (24, 48)	29 (21, 35)
Gender—male	273 (70%)	26 (70%)	24 (50%)	97 (65%)	120 (81%)	6 (100%)
Smoking	60 (30%)	0 (0%)	3 (13%)	22 (25%)	33 (45%)	2 (67%)
Unknown	188	22	25	63	75	3
Diabetes mellitus	14 (4.0%)	1 (2.9%)	1 (2.3%)	8 (5.8%)	4 (3.0%)	0 (0%)
Unknown	36	3	5	13	13	2
Work status						
Student	36 (9.3%)	2 (5.4%)	3 (6.3%)	11 (7.3%)	19 (13%)	1 (17%)
Working	183 (47%)	20 (54%)	18 (38%)	72 (48%)	70 (47%)	3 (50%)
Not working	24 (6.2%)	2 (5.4%)	5 (10%)	8 (5.3%)	8 (5.4%)	1 (17%)
Retired	47 (12%)	4 (11%)	9 (19%)	19 (13%)	15 (10%)	0 (0%)
Unknown	99 (25%)	9 (24%)	13 (27%)	40 (27%)	36 (24%)	1 (17%)
Hand comorbidity	45 (12%)	5 (14%)	1 (2.1%)	16 (11%)	23 (16%)	0 (0%)
Trauma mechanism						
Low energy	373 (96%)	36 (97%)	47 (98%)	146 (97%)	138 (93%)	6 (100%)
High energy	3 (0.8%)	1 (2.7%)	0 (0%)	0 (0%)	2 (1.4%)	0 (0%)
Crush	13 (3.3%)	0 (0%)	1 (2.1%)	4 (2.7%)	8 (5.4%)	0 (0%)
Dominant hand is affected hand	197 (67%)	19 (68%)	24 (63%)	55 (51%)	97 (80%)	2 (100%)
Unknown	93	9	10	43	27	4
Median days from injury to presentation (IQR)	1 (0, 2)	1 (0, 3)	1 (0, 2)	1 (0, 2)	0 (0, 1)	1 (0, 3)
Affected digit						
2	31 (8.0%)	7 (19%)	3 (6.3%)	6 (4.0%)	13 (8.8%)	2 (33%)
3	28 (7.2%)	3 (8.1%)	1 (2.1%)	23 (15%)	0 (0%)	1 (17%)
4	70 (18%)	3 (8.1%)	5 (10%)	51 (34%)	11 (7.4%)	0 (0%)
5	260 (67%)	24 (65%)	39 (81%)	70 (47%)	124 (84%)	3 (50%)
Soft tissue damage	23 (6.0%)	6 (17%)	0 (0%)	6 (4.1%)	11 (7.4%)	0 (0%)
Unknown	3	1	0	2	0	0
Clinically observed angulation	25 (6.5%)	2 (5.6%)	0 (0%)	12 (8.1%)	11 (7.5%)	0 (0%)
Unknown	4	1	0	2	1	0
Rotational deformity	29 (7.6%)	0 (0%)	2 (4.2%)	13 (8.7%)	14 (9.7%)	0 (0%)
Unknown	5	1	0	1	3	0
Displacement of fracture on radiograph (> 2 mm)	157 (40%)	13 (35%)	11 (23%)	55 (37%)	76 (51%)	2 (33%)
Closed fracture reduciton	45 (12%)	1 (2.7%)	2 (4.2%)	21 (14%)	21 (14%)	0 (0%)
Treating specialism						
Trauma surgeon	319 (82%)	32 (86%)	39 (81%)	128 (85%)	115 (78%)	5 (83%)
Plastic surgeon	1 (0.3%)	0 (0%)	0 (0%)	0 (0%)	0 (0%)	1 (17%)
Orthopaedic surgeon	42 (11%)	5 (14%)	6 (13%)	17 (11%)	14 (9.5%)	0 (0%)
ED physician	27 (6.9%)	0 (0%)	3 (6.3%)	5 (3.3%)	19 (13%)	0 (0%)
Referral to hand therapy	42 (11%)	5 (14%)	7 (15%)	17 (11%)	13 (8.8%)	0 (0%)
Unknown	2	0	0	1	1	0
Complications	5 (1.3%)	1 (2.7%)	0 (0%)	3 (2.0%)	1 (0.7%)	0 (0%)

### Analyses of non-operative treatment methods and duration of immobilisation

**Fig. 2:**
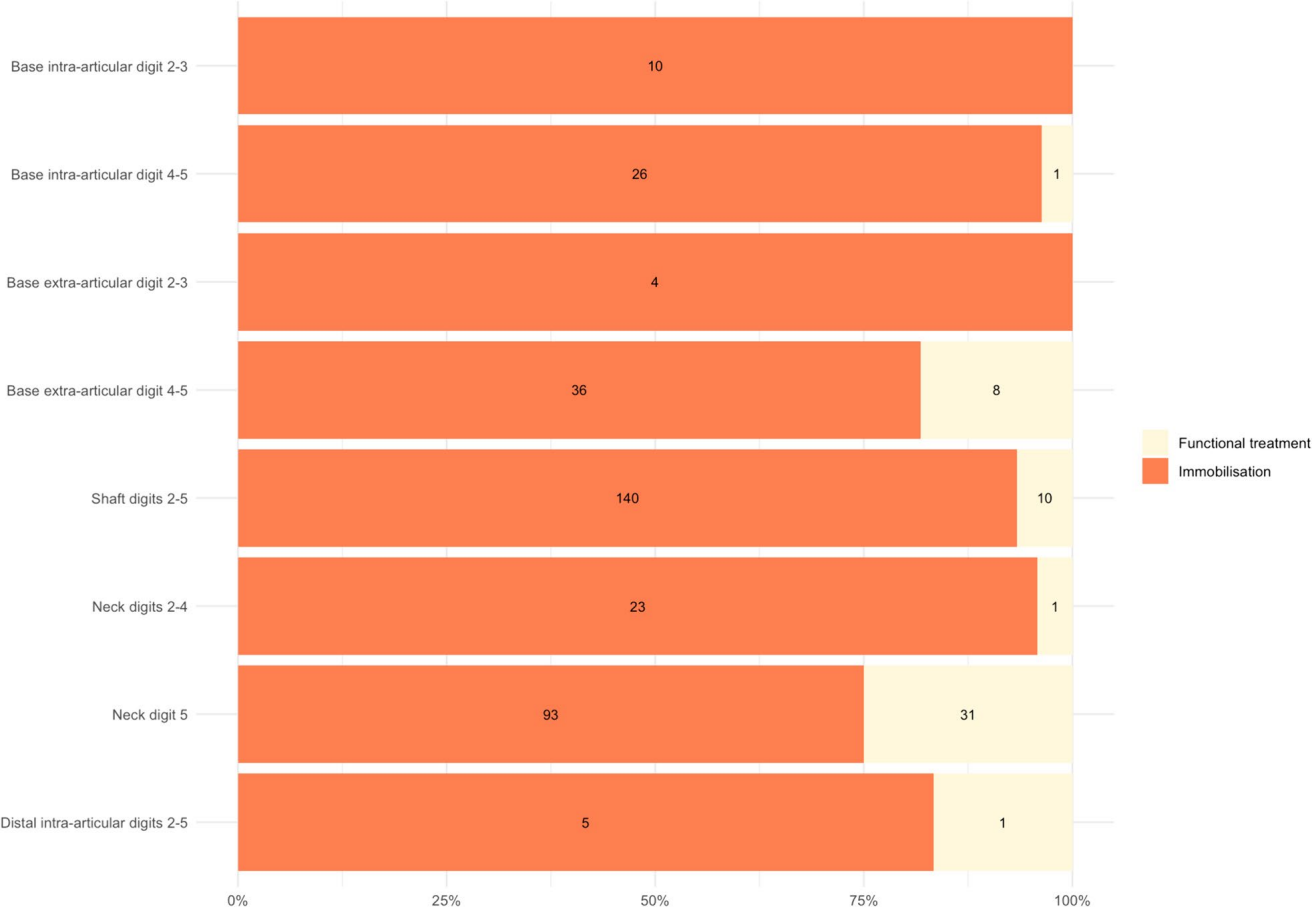
Distribution of treatment methods for the eight most common metacarpal fracture types. The bars show the proportion of patients receiving functional treatment versus immobilisation across different fracture types, with the cumulative total reaching 100%. Absolute fracture numbers corresponding to each treatment method are specified for each fracture type

At the ED, 337 (87%) patients were immobilised, while 52 (13%) received functional treatment. Treatment variation was present in three out of eight fracture groups: shaft fractures (140 patients (93%) received immobilisation), fifth MC neck fractures (93 patients (75%)), and extra-articular base fractures of digits 4 and 5 (40 patients (83%)). For the results of all groups see Fig. 2.

In the three groups exhibiting variation, bivariate analysis showed no of differences in injury characteristics between functional treatment and immobilisation for shaft fractures and base extra-articular fractures of digits 4 and 5. For fifth metacarpal neck fractures, fracture reduction was not performed in any cases of functional treatment (0/32), while it was performed in 22% (21/95) of cases with immobilisation (*p* = 0.004). For the results of all analyses see Table S1A, B and C.

**Fig. 3:**
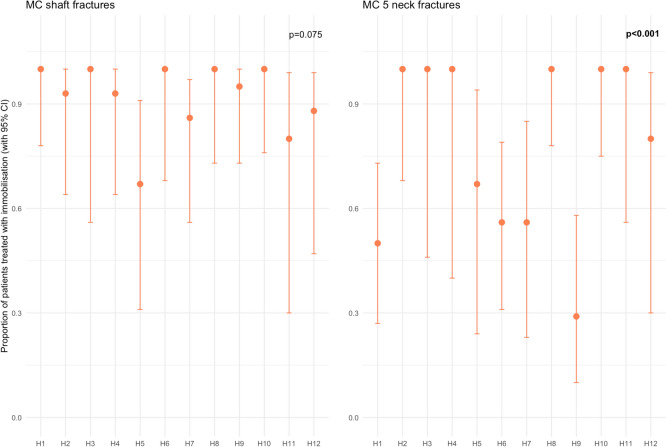
Proportion of patients treated with immobilisation for metacarpal neck fractures across different hospitals. The bars represent the 95% confidence intervals for the proportion treated with immobilisation. Chi-squared test was used to compare immobilisation and hospital setting

Subgroup analyses of shaft fractures and fifth MC neck fractures showed variation in immobilisation versus functional treatment between hospitals ranging from 67% (95% confidence interval (CI) 0.31–0.9) to 100% (95% CI 0.56–1.00), and from 29% (95% CI 0.10–0.58) to 100% (95% CI 0.78–1.00) respectively (Fig. 3).

**Fig. 4:**
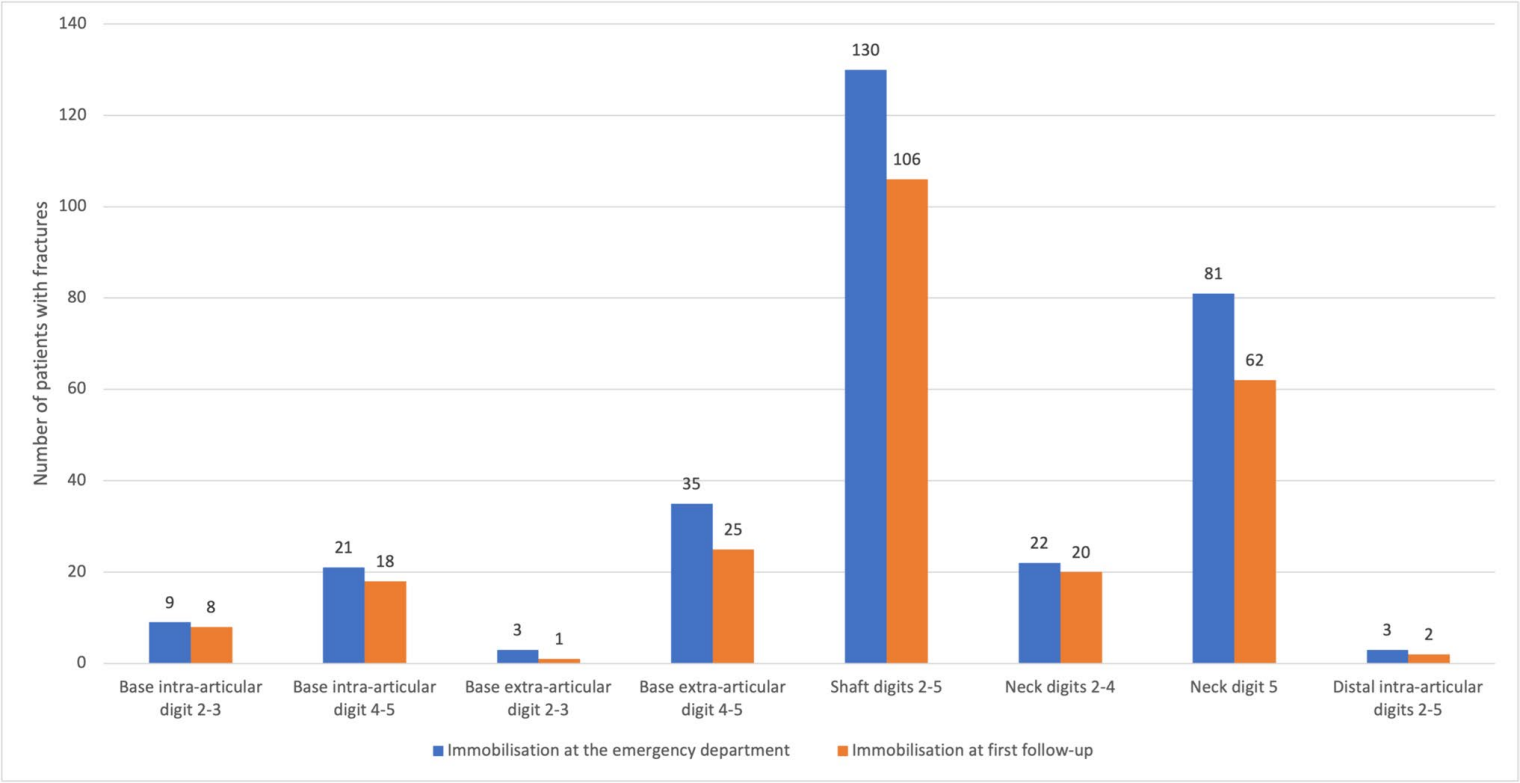
The number of patients who received immobilisation at the emergency department versus at first follow-up for the eight most common fracture types

Out of the initial cohort of 389 patients who presented at the ED, 13 patients received follow-up at a different hospital and 22 patients failed to attend their scheduled follow-up appointments. Therefore, 354 patients were eligible for inclusion in the analysis of treatment at the outpatient clinic. At the first follow-up visit, treatment was changed from immobilisation to functional treatment in 10 out of 43 patients (23%) with extra-articular base fractures of digit 4 and 5, 25 out of 139 patients (18%) with shaft fractures, 19 out of 111 patients (17%) for fifth MC neck fractures. Results for all groups are shown in Fig. 4. The median day of the first follow-up visit was day 8 (IQR: 7–9).

**Fig. 5:**
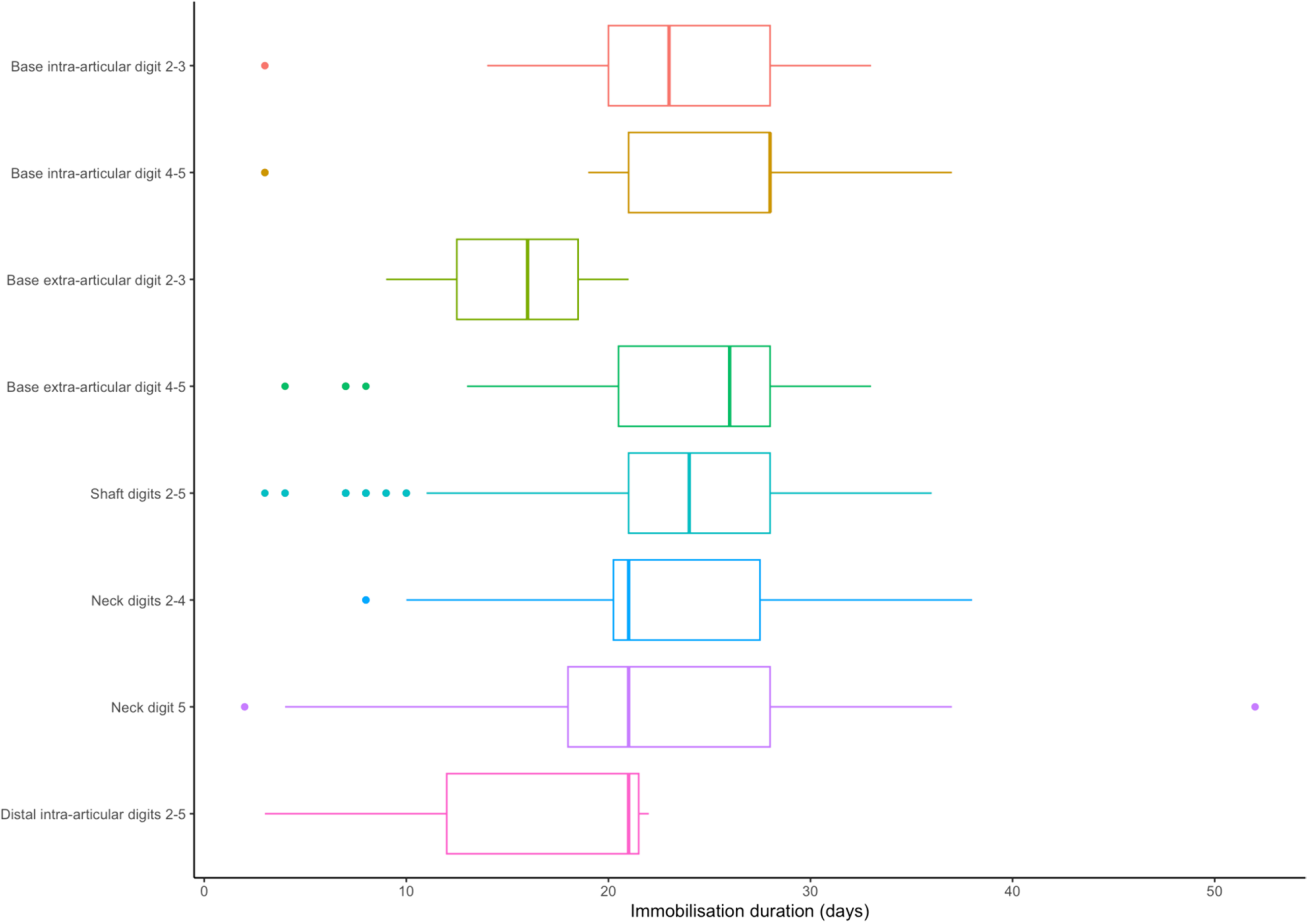
Immobilisation duration in days for different metacarpal fracture types

The median duration of immobilisation for all fractures was 23 days (IQR: 20–28), with no differences in the duration of immobilisation between fracture categories (*p* = 0.10) (Fig. 5). Multivariable linear regression showed that hospital was highly predictive of duration of immobilisation (*p* < 0.001), whereas individual patient and injury characteristics appeared not predictive of this duration (Table 2).

**Table 2:** Multivariable linear regression to assess the impact hospital variation on the length of immobilisation for 303 patients who were immobilised

	Coefficient	95% CI^a^	p value
Hospital			** < 0.001**
H1	ref		
H2	− 2.3	− 6.5, 1.9	
H3	5.4	0.73, 10	
H4	− 0.79	− 5.0, 3.4	
H5	− 3.2	− 7.9, 1.5	
H6	− 6.4	− 11, − 2.1	
H7	− 1.5	− 5.8, 2.9	
H8	− 1.7	− 5.7, 2.3	
H9	1.4	− 2.4, 5.2	
H10	2.2	− 1.7, 6.1	
H11	4.4	− 0.76, 9.5	
H12	− 2.5	− 7.4, 2.4	
Fracture group			0.4
Base intra-articular digit 2–3	ref		
Base intra-articular digit 4–5	1.9	− 4.4, 8.1	
Base extra-articular digit 2–3	− 6.4	− 17, 4.1	
Base extra-articular digit 4–5	0.98	− 4.9, 6.8	
Shaft digits 2–5	1.1	− 4.3, 6.6	
Neck digits 2–4	0.05	− 6.1, 6.2	
Neck digit 5	− 0.86	− 6.5, 4.7	
Distal intra-articular digits 2–5	− 5.7	− 16, 4.7	
Clinically observed angulation	− 0.89	− 4.5, 2.7	0.6
Rotational deformity	0.21	− 3.3, 3.8	> 0.9
Dislocation on radiograph (> 2 mm)	1.3	− 0.82, 3.5	0.2
Closed fracture reduction	0.38	− 2.7, 3.5	0.8

A *p* value of < 0.05 was taken as a threshold of statistical significance and is printed in bold

Results are presented as regression coefficients with corresponding 95% confidence intervals. A positive score indicates that the factor is associated with a longer immobilization duration

^a^*CI* confidence interval

### Patient-reported outcomes in relation to treatment method and duration of immobilisation

**Fig. 6:**
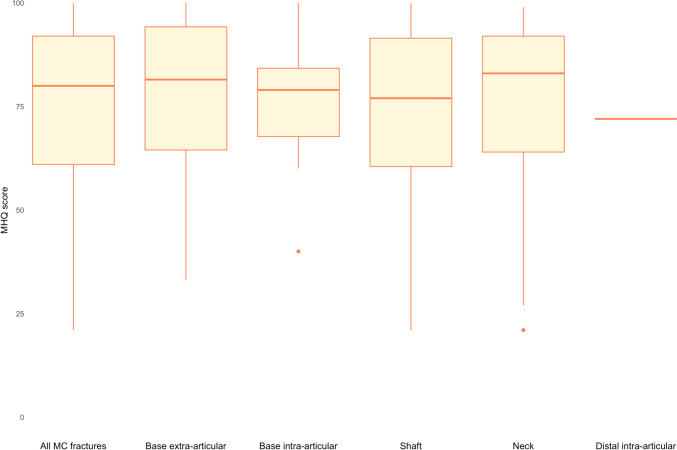
Michigan Hand Outcomes Questionnaire scores for all metacarpal fractures and for the five most common fracture types, three months post-trauma. The maximum score is 100 points, which is the best possible score: a lower the score indicates a worse overall hand function

The median MHQ score was 80 (IQR: 61–92), with no differences between the five fracture categories (*p* > 0.9). The results for all fracture categories are summarized in Fig. 6. Among shaft fractures, the median MHQ score following functional treatment was 87 (IQR: 84–89), whereas after immobilisation it was 76 (IQR: 60–92) (*p* = 0.4). For fifth MC neck fractures, the MHQ scores were 92 (IQR: 69–94) and 80 (IQR: 69–91) respectively (*p* = 0.4) (Table 3). Patients with MC shaft fractures immobilised for less than 21 days had significantly higher MHQ scores compared to those immobilised for 21 days or more, with a median MHQ score of 83 (IQR:76–100) and 71 (IQR: 57–89), respectively (*p* = 0.026) (see Table 4).

**Table 3:** MHQ scores of functional treatment compared to immobilisation for all metacarpal fractures, metacarpal shaft fractures, and fifth metacarpal neck fractures

	Overall	Functional treatment	Immobilisation	*p* value^a^
All MC fractures	n = 113	n = 14	n = 99	
Median MHQ (IQR)	80 (61, 92)	87 (67, 94)	78 (61, 91)	0.4
MC shaft fractures	n = 51	n = 2	n = 49	
Median MHQ (IQR)	77 (61, 92)	87 (84, 89)	76 (60, 92)	0.4
Fifth MC neck fractures	n = 24	n = 7	n = 17	
Median MHQ (IQR)	83 (69, 92)	92 (69, 94)	80 (69, 91)	0.4

*MHQ* Michigan Hand outcomes Questionnaire, *MC* metacarpal, *IQR* interquartile range

^a^Wilcoxon rank sum test

**Table 4:** MHQ scores of fractures immobilised < 21 days versus ≥ 21 days for all metacarpal fractures, metacarpal shaft fractures, and fifth metacarpal neck fractures

	Immobilisation < 21 days	Immobilisation ≥ 21 days	*p* value^a^
All MC fractures	n = 25	n = 72	
Median MHQ (IQR)	80 (72, 96)	78 (60, 90)	0.2
MC Shaft fractures digits 2–5	n = 11	n = 36	
Median MHQ (IQR)	83 (76, 100)	71 (57, 89)	**0.026**
MC neck fractures digit 5	n = 6	n = 11	
Median MHQ (IQR)	78 (70, 82)	90 (74, 93)	0.3

A *p* value of < 0.05 was taken as a threshold of statistical significance and is printed in bold

*MHQ* Michigan Hand outcomes Questionnaire, *MC* metacarpal, *IQR* interquartile range

^a^Wilcoxon rank sum test

## Discussion

This multicentre snapshot study investigated non-operative treatment methods and the immobilisation duration for MC fractures, exploring potential practice variations and evaluating patient-reported outcomes. Our findings indicate that plaster immobilisation is the predominant non-operative treatment method for all MC fracture categories, including fifth MC neck fractures, which contrasts with the Dutch national guideline recommending functional treatment [11]. The variation in treatment approaches between hospitals may explain this discrepancy. Regarding immobilisation duration, we found that most fracture categories experienced prolonged immobilisation (21 days or more). There appears to be practice variation between hospitals in immobilisation duration, with hospital setting being independently associated with prolonged immobilisation. Analysis of patient-reported outcomes indicated that prolonged immobilisation duration was associated with worse MHQ scores for patients with MC shaft fractures.

Strengths of our study include its comprehensive overview of non-operative treatment methods for all MC fractures across multiple centres, providing a good understanding of current non-operative treatment approaches. Based on the results, we recommend revising or optimizing local hospital protocols for fifth MC neck fractures. This will lead to improved patient care and reduces costs. Despite its strengths, the study has some limitations. First, the three-month study period limits the ability to analyse less common subgroups due to small sample sizes. Additionally, analyses of the relation between practice variation and patient-reported outcome measures were limited by the small sample size. Second, while the regression analysis of factors associated with immobilisation duration corrected for injury-related factors and hospital setting, other patient-related variables such as age, sex, and pain may impact the immobilisation duration, indicating unexplored variability. These patient-related factors were excluded to prevent model overfitting, given the sample size. Third, although our study showed significantly higher MHQ scores for shaft fractures immobilised for less than 21 days compared to those immobilised for 21 days or more, the 12-point difference in median MHQ score (the MHQ score was 83 for less than 21 days and 71 for 21 days or more) may lack clinical significance. The minimally clinically important difference (MCID) (minimal effect that would be meaningful to patients) and the minimally important difference (MID) (minimal difference that reflects a true improvement) for the MHQ score after hand fractures are unknown. Further studies should investigate the MCID and MID of the MHQ score after hand fractures to improve the interpretation of the findings of future studies.

For patients with shaft fractures, who were primarily treated with immobilisation in most cases (93%), our results suggest that treatment selection is unlikely influenced by injury characteristics, as no differences were observed between functional treatment and immobilisation. Additionally, no meaningful differences in treatment selection were observed between hospitals. The median duration of immobilisation was 24 days (IQR: 21–28), which contrasts with a recently published randomized showing similar outcomes between non-operative treatment with unrestricted mobilization and operative treatment with screws for displaced spiral and/or oblique shaft fractures of digits two to five. Costs and sick leave were substantially higher in the operative group [12]. These results imply that more patients could potentially benefit from immediate functional treatment, which has the potential to reduce costs, limit hospital visits, improve resource allocation in hospitals and reduce work absenteeism [5].

For fifth MC neck fractures, 58% of patients received immobilisation at the ED, with only 17% transitioning to functional treatment during the initial follow-up visit. The median duration of immobilisation was 21 days (IQR: 19–28), indicating a tendency toward prolonged immobilisation. This contradicts evidence-based treatment recommendations advocating early functional treatment for fifth MC neck fractures [7, 13–15]. The Dutch guideline for hand fractures, published in 2019, advises early functional treatment, preferably within one week, unless patients prefer immobilisation over functional treatment due to pain [11]. A gap appears between the guidelines and clinical practice, possibly due to patient preferences and practical issues in implementing evidence-based recommendations. Additionally, fracture reduction may influence the choice, as bivariate analysis showed an association between fracture reduction and immobilisation. However, it is questionable whether fracture reduction was always indicated, as the guideline recommends it only for fractures with more than 70 degrees of angulation. Literature suggests a trend toward treating these fractures with functional bracing regardless of palmar angulation, except in patients with exceptional demands or other fracture deformities [16]. Our results showed that the percentage of immobilisation for fifth MC neck fractures varied between hospitals from 29 to 100%, suggesting outdated hospital protocols may contribute largely to this variation. Increased adherence to guidelines is necessary for optimal patient treatment and cost-effectiveness.

Regarding shaft fractures, better MHQ scores were observed after functional treatment (median 87; IQR: 67–94) compared to immobilisation (median 78; IQR: 61–91). MHQ scores were significantly higher after short immobilisation compared to long immobilisation. The results should be interpreted with caution due the small sample size and the potential incomparability in patient and fracture characteristics between these groups. A systematic review published in 2022 assessing different treatment modalities for shaft fractures found three studies comparing two non-operative treatment methods, none of which included patient-reported outcomes [17–20]. They concluded that there is no evidence to support any one treatment over another. One RCT published in 2022, comparing non-operative treatment (immediate mobilization) and surgery showed no difference in Disabilities of the Arm, Shoulder and Hand (DASH) scores between the two groups [13]. For fifth MC neck fractures, MHQ scores after functional treatment were 92 (IQR 69, 94) compared to 80 (IQR 69, 91) after immobilisation, consistent with a recent systematic review showing an equal effect or an advantage for using buddy taping over casing for closed fifth MC neck fractures [21].

In conclusion, this study highlights significant practice variations in the non-operative treatment of MC fractures across Dutch hospitals, particularly in immobilisation methods and duration. Variations were noted in the treatment of fifth MC neck fractures, with some hospitals adhering to the guideline advocating functional treatment and others not. The data suggest that prolonged immobilisation, especially for MC shaft fractures, may be associated with worse patient-reported outcomes. These findings underscore the need for adherence to treatment protocols and emphasize functional treatment to potentially improve patient outcomes and cost-effectiveness.

## Supplementary Information

Below is the link to the electronic supplementary material.

Supplementary file1 (DOCX 13 KB)

Supplementary file2 (DOCX 13 KB)

Supplementary file3 (DOCX 13 KB)

## Data Availability

Data cannot be shared openly to protect study participant privacy. Original data are stored using Castor EDC.
