# Supplementary material for: Non-operative treatment of metacarpal fractures and patient-reported outcomes: a multicentre snapshot study
Source: Eur J Trauma Emerg Surg. 2024 Sep 23;50(5):2399–409. doi: 10.1007/s00068-024-02659-9 (PMC11599336; doi:10.1007/s00068-024-02659-9)
Supplement: Supplementary file 1 — Supplementary file1 (DOCX 13 KB) [file 68_2024_2659_MOESM1_ESM.docx]

| **Table S1A. Injury characteristics metacarpal shaft fractures** | | | |
| --- | --- | --- | --- |
|  | Functional treatment  N = 10 | Immobilization  N = 140 | *p*-value*^1^* |
| Affected digit |  |  | 0.8 |
| 2 | 0 (0%) | 6 (4.3%) |  |
| 3 | 1 (10%) | 22 (16%) |  |
| 4 | 5 (50%) | 46 (33%) |  |
| 5 | 4 (40%) | 66 (47%) |  |
| Type of fracture |  |  | 0.8 |
| Multi-fragmentary | 0 (0%) | 3 (2.1%) |  |
| Oblique | 7 (70%) | 104 (74%) |  |
| Transverse | 3 (30%) | 33 (24%) |  |
| Clinically observed angulation | 1 (10%) | 11 (8.0%) | 0.6 |
| Unknown | 0 | 2 |  |
| Clinically observed rotational deformity | 0 (0%) | 13 (9.4%) | 0.6 |
| Unknown | 0 | 1 |  |
| Dislocation on radiograph (>2mm) | 2 (20%) | 53 (38%) | 0.3 |
| Closed fracture reduction | 2 (20%) | 19 (14%) | 0.6 |
| *^1^* Fisher’s exact test | | | |
