# Supplementary material for: Non-operative treatment of metacarpal fractures and patient-reported outcomes: a multicentre snapshot study
Source: Eur J Trauma Emerg Surg. 2024 Sep 23;50(5):2399–409. doi: 10.1007/s00068-024-02659-9 (PMC11599336; doi:10.1007/s00068-024-02659-9)
Supplement: Supplementary file 2 — Supplementary file2 (DOCX 13 KB) [file 68_2024_2659_MOESM2_ESM.docx]

| **Table S1B. Injury characteristics fifth metacarpal neck fractures** | | | |
| --- | --- | --- | --- |
|  | Functional treatment,  N = 32 | Immobilization,  N = 95 | *p*-value*^1^* |
| Clinically observed angulation | 0 (0%) | 10 (11%) | 0.064 |
| Unknown | 0 | 1 |  |
| Clinically observed rotational deformity | 2 (6.5%) | 12 (13%) | 0.5 |
| Unknown | 1 | 2 |  |
| Dislocation on radiograph (>2mm) | 14 (44%) | 59 (62%) | 0.069 |
| Closed fracture reduction | 0 (0%) | 21 (22%) | **0.004** |
| *^1^* Fisher’s exact test; Pearson’s Chi-squared test | | | |
