# Supplementary material for: Non-operative treatment of metacarpal fractures and patient-reported outcomes: a multicentre snapshot study
Source: Eur J Trauma Emerg Surg. 2024 Sep 23;50(5):2399–409. doi: 10.1007/s00068-024-02659-9 (PMC11599336; doi:10.1007/s00068-024-02659-9)
Supplement: Supplementary file 3 — Supplementary file3 (DOCX 13 KB) [file 68_2024_2659_MOESM3_ESM.docx]

| **Table S1C. Injury characteristics metacarpal base extra-articular fractures of digits four and five** | | | |
| --- | --- | --- | --- |
| **Characteristic** | Functional treatment  N = 8 | Immobilization  N = 36 | *p*-value*^1^* |
| Clinically observed angulation | 0 (0%) | 0 (0%) | >0.9 |
| Clinically observed rotational deformity | 0 (0%) | 2 (5.6%) | >0.9 |
| Dislocation on radiograph (>2mm) | 1 (13%) | 10 (25%) | 0.7 |
| Closed fracture reduction | 0 (0%) | 2 (5.6%) | >0.9 |
| *^1^* Fisher’s exact test | | | |
